# Agricultural ditches and stream networks are overlooked hotspots of carbon emissions

**DOI:** 10.1093/nsr/nwaf111

**Published:** 2025-03-25

**Authors:** Lei Zhou, Yongqiang Zhou, José R Paranaíba, Mike Peacock, Erik Jeppesen, David P Hamilton

**Affiliations:** State Key Laboratory of Soil and Sustainable Agriculture, Institute of Soil Science, Chinese Academy of Sciences, China; Taihu Laboratory for Lake Ecosystem Research, State Key Laboratory of Lake and Watershed Science for Water Security, Nanjing Institute of Geography and Limnology, Chinese Academy of Sciences, China; University of Chinese Academy of Sciences, China; Department of Ecology, Radboud Institute for Biological and Environmental Sciences, Radboud University, the Netherlands; Department of Aquatic Sciences and Assessment, Swedish University of Agricultural Sciences, Sweden; Department of Geography and Planning, School of Environmental Sciences, University of Liverpool, UK; Department of Ecoscience and Centre for Water Technology (WATEC), Aarhus University, Denmark; Sino-Danish Centre for Education and Research, China; Institute for Ecological Research and Pollution Control of Plateau Lakes, School of Ecology and Environmental Science, Yunnan University, China; Griffith University, Australian Rivers Institute, Australia

**Figure 1.**
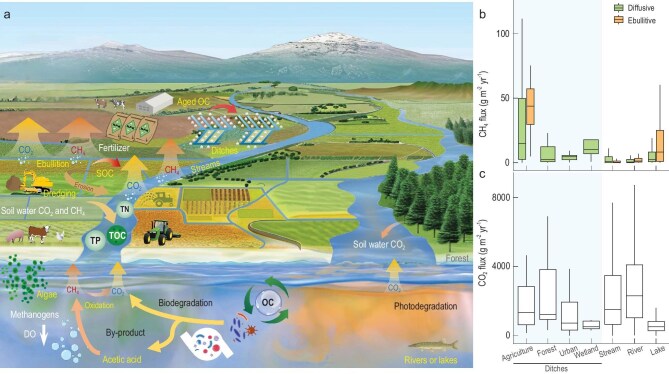
Agricultural ditches and streams as overlooked hotspots of carbon processing. (a) A schematic illustration depicting how agricultural practices enhance soil organic matter (SOM) input into waterways, alter water chemistry, and promote the outgassing of carbon dioxide (CO_2_) and methane (CH_4_) from ditches, streams, and downstream-connected river or lake ecosystems. (b) Boxplots showing CH_4_ flux variations among different ditch types—agricultural (*n*_diffusive_ = 41, *n*_ebullitive_ = 9), forest (*n* = 24), urban (*n* = 10), wetland (*n* = 5)—as well as streams (*n*_diffusive_ = 321, *n*_ebullitive_ = 27), rivers (*n*_diffusive_ = 157, *n*_ebullitive_ = 20), and lakes (including impoundments, *n*_diffusive_ = 631, *n*_ebullitive_ = 144). (c) Boxplots showing CO_2_ flux variations among different ditch types—agricultural (*n* = 40), forest (*n* = 26), urban (*n* = 12), wetland (*n* = 5)—as well as streams (*n* = 311), rivers (*n* = 170), and lakes (including impoundments, *n* = 7250). Abbreviations: DO (dissolved oxygen), OC (organic carbon), SOC (soil organic carbon), TP (total phosphorus), TOC (total organic carbon), TN (total nitrogen), and *n* (number of samples). Carbon flux data were obtained from references: [15] for ditches, [16] for streams and rivers, and [17] for lakes (including impoundments). The stream and river categories represent Strahler stream orders 1–2 and ≥5, respectively, as classified in the Global River Methane Database of concentrations and fluxes (using non-targeted sites). The data used in Fig. 1b–c is available at https://doi.org/10.6084/m9.figshare.28560338.v2.

In many agricultural regions, ditch-stream networks play a critical role in preventing waterlogging, enhancing soil aeration, and channeling water to crops in areas with insufficient rainfall, promoting productivity. These networks facilitate the lateral transfer of water, energy, and materials between upstream agricultural lands and downstream rivers or lakes (Fig. 1a). Ditches typically form a dense network across agricultural landscapes, and cover an area of ∼5.4 × 10^4^ km^2^ [1], which accounts for <2% of the total global lake area (3.2 × 10^6^ km^2^). Ditches generally occupy 3%–5% of the agricultural landscape and contribute 15%–50% of annual carbon emissions [2,3]. For example, in Great Britain, the total length of ditches is estimated at 60 400 km—more than twice the combined length of streams and rivers (26 700 km) [1]. Similarly, the Netherlands has a high density of agricultural ditches (∼300 000 km) [4], while Sweden's ditch network is even more extensive, spanning 756 000 km and covering ∼690 km^2^ [5], which is <2% of Sweden's lake area (∼38 000 km^2^). Moreover, ditches account for 84% of Sweden's national methane (CH_4_) emissions from flooded lands (34 000 t CH_4_ yr^−^^1^), including reservoirs and ponds [5]. However, the mechanisms driving organic carbon transformation and carbon emissions in intensively managed ditch-stream systems remain underexplored and are often poorly quantified, particularly regarding internal transformation processes.

Agricultural inputs modify organic matter (OM) composition and microbial metabolic functions, enhancing carbon dioxide (CO_2_) and CH_4_ production in ditches and streams (Fig. 1a). Farming practices such as intensive tillage and the application of biochar or organic fertilizers can increase the proportion of large soil aggregates and overall organic content [6]. Deforestation and agricultural expansion expose deeper soil horizons, resulting in the mobilization of older OM (bulk ∆^14^C value of −179 ± 16‰; ^14^C age: ∼1.5 kyr) that is more biologically reactive in these disturbed catchments compared to pristine forest catchments [7]. This aged OM is rich in energy content and chemically diverse, with notably higher proportions of nitrogen- and sulfur-containing compounds [7]. Furthermore, agricultural runoff containing fertilizers may stimulate primary productivity and accelerate OM decomposition, increasing the proportion of autochthonous OM from algae and microbes. These processes lead to higher concentrations of organic acids and heteroatomic organic compounds in ditches and streams, enhancing microbial activity and promoting CO_2_ and CH_4_ production.

The bioavailability of dissolved organic carbon (DOC) in agricultural streams and ditches can reach up to 40% [7], indicating enhanced production of low-molecular-weight organic acids (e.g. acetic acid) through microbial degradation. Elevated organic acid content directly supports methanogenesis by supplying essential precursors for CH_4_ production under anaerobic conditions (Fig. 1a). In the UK, excess fine sediment delivery intensifies headwater stream CH_4_ emissions (from 0.2 to 0.7 mmol CH_4_ m^−2^ d^−1^) by increasing streambed organic carbon content (from 23 to 100 g m^−2^), reducing bed permeability, and restricting oxygen flow, which limits CH_4_ oxidation [8]. Terrestrial environments have been shown to directly transport 81%–90% of CO_2_ from soil leaching (e.g. CO_2_ derived from SOM degradation or root respiration bound in the unsaturated soil zone) directly to ditches and small stream systems with discharge <0.01 m^3^ s^−1^ in North America [9].

CH_4_ is emitted in aquatic systems through three primary pathways: diffusion, ebullition (bubble release), and plant-mediated transport. Ebullition can significantly contribute total ditch emissions, accounting for up to 58% (mean: 145 ± 194 mg m^−2^ day^−1^) of greenhouse gas emissions and up to 80% of total CH_4_ emissions in some Dutch agricultural drainage ditches situated on mineral soils (clay atop peat) [10,11]. However, other studies report ebullition as a minor contributor [12], likely due to differences in environmental conditions (e.g. sediment organic matter content, temperature, and water depth) and methodological variations (e.g. timing and duration of measurements). CH_4_ oxidation by methanotrophic microorganisms can mitigate emissions, reducing up to 76.4% ± 12.0% (or 70 mg CH_4_ m^−2^ d^−1^) of potential emissions from canals draining tropical peatlands [13], with even greater reductions when canals are vegetated. The proportion of CH_4_ oxidized is influenced by factors such as dissolved oxygen levels, vegetation, ditch water depth, and sediment substrate (e.g. the availability of electron acceptors like NO^3−^-N, Mn, Fe, and SO_4_^2−^) [10,11].

Agricultural ditch-stream systems generally exhibit a high surface-area-to-volume ratio and receive high inputs of labile OM, nutrients, and dissolved CO_2_ and CH_4_ produced in adjacent agricultural landscapes [1,9]. Ditches are typically slow-flowing waters that promote sediment accumulation, emergent vegetation, and anoxic conditions, all of which favor CH_4_ production and emissions. These factors collectively promote ditches as hotspots for CO_2_ and CH_4_ emissions, often exceeding emissions from other inland water bodies by significant margins. Although lower-order streams (stream orders 1–4) occupy a relatively small area within river networks, they disproportionately influence overall carbon emissions, accounting for up to 70% of total CO_2_ emissions from streams and rivers [14].

Globally, ditches emit 30.0 Tg C yr^−1^ (95% CI: 22.4–37.7) as CO_2_ and 0.03 Tg N yr^−1^ (95% CI: 0.01–0.05) as N_2_O [15], along with 3.5 Tg CH_4_ yr^−1^—equivalent to 0.2%–3% of global anthropogenic CH_4_ emissions [1]. CH_4_ emissions from ditches are positively correlated with temperature and reach their highest levels in eutrophic ditches [1]. The annual emissions of CH_4_ per unit area in agricultural ditches (median: 18.3 and 44.7 g CH_4_ m^−2^ yr^−1^ for diffusive and ebullitive fluxes, respectively) are significantly higher than those in other ditch types, as well as in streams (median: 4.4 and 1.3 g CH_4_ m^−2^ yr^−1^), rivers (median: 2.7 and 3.3 g CH_4_ m^−2^ yr^−1^), and lakes (including impoundments, median: 2.9 and 10.3 g CH_4_ m^−2^ yr^−1^) (Fig. 1b). Globally, CO_2_ emissions from ditches do not exhibit significant patterns across different land use types, climate zones, trophic states, soil types, hydrological regimes, or the presence of vegetation. The lack of a response of CO_2_ to broad climate zones and land use categories may be because local-scale temporal, environmental, or management conditions may have a greater impact [15]. The annual CO_2_ emissions in agricultural ditches exceed those from other types of ditches and lakes (including impoundments) but are lower than those from streams and rivers (Fig. 1c). This discrepancy is closely linked to the higher flow velocity (i.e. greater water–atmosphere gas exchange) and faster water renewal cycles in streams and rivers compared to ditches.

Notably, in the North China Plain, CH_4_ emissions (333 μmol m^−2^ h^−1^) from ditch systems can be four times higher than the global average for inland water bodies (88 μmol m^−2^ h^−1^), while CH_4_ concentrations (14.8 μmol L^−1^) exceed the global river and stream average (1.35 μmol L^−1^) by more than 10-fold [2]. Additionally, these agricultural ditches exhibit CH_4_ emissions 12 times higher and CO_2_ emissions 5 times higher than those of the rivers they connect to [2]. Although ditches in the North China Plain cover only 3.3% of farmland area, their emissions (26.6 Gg CO_2_-equivalent yr^−1^) account for ∼30% of the net greenhouse gas emissions (∼88 Gg CO_2_-equivalent yr^−1^) [2], highlighting the disproportionate contribution of ditches and streams to carbon emissions. In Sweden, total carbon emissions from low-order streams alone (2.7 Tg C yr^−1^) are comparable to the estimated CO_2_ emissions from all inland waters nationally (2.6 Tg C yr^−1^: lakes 1.8 Tg C yr^−1^; streams 0.8 Tg C yr^−1^) [14]. Ditches alone emit 28 700 t CH_4_ yr^−1^, compared to ∼1700 t CH_4_ yr^−1^ for ponds and 3400 t CH_4_ yr^−1^ for reservoirs in Sweden [5]. These stream carbon emissions represent 21% of Sweden's net atmospheric carbon uptake from land use, land-use change, and forestry (12.8 Tg C yr⁻^1^ in 2014) [14]. Despite covering <4% of the total catchment area, ditches accounted for 31% of total N_2_O emissions in an agricultural catchment in England [15]. CH_4_ emissions from ditches in the Netherlands are estimated at 0.09 Tg CH_4_ yr⁻^1^ (3.0 Tg CO_2_-equivalent yr⁻^1^), representing ∼16% of the country's annual CH_4_ emissions [4].

Despite being hotspots of regional CO_2_ and CH_4_ emissions (Fig. 1a), agricultural ditches and stream networks suffer from a lack of reliable data, particularly on CH_4_ ebullition, with adequate spatial and temporal coverage. Such data are essential for scaling emissions from local landscapes to global estimates. Low-order ditch-stream systems are not only underrepresented in CO_2_ and CH_4_ sampling efforts but also lack consistent discharge measurements. Their highly complex connectivity across the terrestrial-water interface further complicates remote sensing assessments of total ditch coverage.

A comprehensive understanding of the coupled mechanisms governing OM composition, microbial diversity and metabolism, and carbon emissions in these agricultural networks is crucial for accurately assessing regional and global carbon fluxes and designing effective agricultural drainage systems that mitigate carbon emissions. For example, agricultural ditches are frequently dredged, making the management of DOC-rich sediment essential for controlling emissions. However, this aspect is often omitted from most carbon budgets. Ditch dredging has been shown to reduce CH_4_ and CO_2_ emissions by ∼35% over the long term [11]. In intensive farming areas, ditches are often artificially modified to facilitate rapid water drainage, acting as conduits for transporting agricultural runoff to downstream ecosystems. Revegetation and riparian protection around ditches and lower-order streams, as well as water level manipulation, could significantly influence hydrology while mitigating and preventing emissions from high-emission zones.

Further research, including field studies and modeling approaches, is needed to better quantify the extent of ditch-stream networks and improve our understanding of carbon composition and dynamics. Key knowledge gaps include the lack of data on diel emission patterns, insufficient estimates of ditch areas affected by wet-dry seasonal cycles, as well as the limited spatial and temporal sampling coverage in agricultural ditch-stream systems. Additionally, gaps remain in direct and indirect assessments of CO_2_, CH_4_, and N_2_O emissions used to determine emission rates, as well as the underrepresentation of ebullition in emission estimates. Furthermore, context-dependent emission factors (e.g. soil type, trophic states) of ditches and small streams require further investigation to refine global carbon budgets.
